# Stabilization of Multicationic Redox Chemistry in Polyanionic Cathode by Increasing Entropy

**DOI:** 10.1002/advs.202202082

**Published:** 2022-07-01

**Authors:** Huangxu Li, Ming Xu, Huiwu Long, Jingqiang Zheng, Liuyun Zhang, Shihao Li, Chaohong Guan, Yanqing Lai, Zhian Zhang

**Affiliations:** ^1^ School of Metallurgy and Environment Engineering Research Center of the Ministry of Education for Advanced Battery Materials Hunan Provincial Key Laboratory of Nonferrous Value‐Added Metallurgy Central South University Changsha 410083 P. R. China; ^2^ Department of Chemistry City University of Hong Kong Kowloon Hong Kong 999077 P. R. China; ^3^ School of Chemistry Xi'an Jiaotong University Xi'an 710049 P. R. China; ^4^ University of Michigan−Shanghai Jiao Tong University Joint Institute Shanghai Jiao Tong University Shanghai 200240 P. R. China

**Keywords:** cathode materials, high entropy, multiple redox reactions, polyanionic materials, sodium‐ion batteries

## Abstract

Polyanionic compounds have large compositional flexibility, which creates a growing interest in exploring the property limits of electrode materials of rechargeable batteries. The realization of multisodium storage in the polyanionic electrodes can significantly improve capacity of the materials, but it often causes irreversible capacity loss and crystal phase evolution, especially under high‐voltage operation, which remain important challenges for their application. Herein, it is shown that the multisodium storage in the polyanionic cathode can be enhanced and stabilized by increasing the entropy of the polyanionic host structure. The obtained polyanionic Na_3.4_Fe_0.4_Mn_0.4_V_0.4_Cr_0.4_Ti_0.4_(PO_4_)_3_ cathode exhibits multicationic redox property to achieve high capacity with good reversibility under the high voltage of 4.5 V (vs Na/Na^+^). Exploring the underlying mechanism through operando characterizations, a stable trigonal phase with reduced volume change during the multisodium storage process is disclosed. Besides, the enhanced performance of the HE material also derives from the synergistic effect of the diverse TM species with suitable molarity. These results reveal the effectiveness of high‐entropy concept in expediting high‐performance polyanionic cathodes discovery.

## Graphical abstract

A high‐entropy Na superionic conductor material Na_3.4_Fe_0.4_Mn_0.4_V_0.4_Cr_0.4_Ti_0.4_(PO_4_)_3_ (HE‐NASICON) is synthesized. It shows that the high entropy effect helps to stabilize the crystal structure and suppress unfavorable phase transition, thus leading to enhanced cyclability. Besides, the enhanced performance also derives from the synergistic effect of the various transition metals with equal molarity.

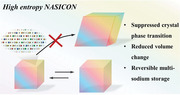

## 1 Introduction

The quick‐emerging paradigm of renewable energy development and use of rechargeable Li‐ion batteries on a large scale are being challenged by the scarcity of lithium sources and uneven geographical distribution.^[^ ^1^, ^2^, ^3^, ^4^ ^]^ In this context, sodium‐ion batteries (SIBs) technology is an attractive option due to the low cost, abundant, and environmentally friendly sodium resources. Moreover, it can be easily and rapidly replicated from Li‐ion battery technologies regarding industrial and commercial processes.^[^ ^5^, ^6^, ^7^, ^8^ ^]^ Major obstacles of using SIBs technology for real applications include the low capacity and unsatisfactory electrochemical stability, which are directly determined by the properties of cathodes in SIBs. Through comparative studies, sodium superionic conductor (NASICON) materials have received considerable interests due to the three‐dimensional (3D) open framework, compositional diversity, and exceptional Na‐ion mobilities.^[^ ^9^, ^10^ ^]^ Recently, researchers incorporate different redox centers into NASICON materials to ensure multiple Na‐ions transfer and high capacities of cathodes, for example Na_3_VCr(PO_4_)_3_,^[^ ^11^ ^]^ Na_4_VMn_0.5_Fe_0.5_(PO_4_)_3_,^[^ ^12^ ^]^ and Na_4_MnV(PO_4_)_3_^[^ ^13^, ^14^ ^]^ etc. Nevertheless, the multi‐Na‐ions intercalation/deintercalation process, which is key to improve capacity of NASICON materials, causes a series of issues, including large volume change, high‐capacity irreversibility, and even irreversible crystal phase evolution (monoclinic → rhombohedral) under high voltage operation (>3.8 V vs Na/Na^+^).^[^ ^15^, ^16^ ^]^ These issues greatly degrade cycling stability of the multiredox reaction and plague the implementation of NASICON cathodes in practical cell.

High‐entropy (HE) alloys have stimulated increasing interest ever since the report of the first example in 2004, mostly ascribed to their high strength and ductility paired with high fracture toughness, fatigue resistance, and creep resistance.^[^ ^17^, ^18^, ^19^, ^20^ ^]^ The evolved HE oxides (HEO),^[^ ^18^, ^21^, ^22^, ^23^ ^]^ dichalcogenides,^[^ ^24^ ^]^ hexacyanometalates,^[^ ^25^ ^]^ etc., also demonstrate unique properties for various applications including catalysis and energy storage. For instance, an O3‐type HEO material NaNi_0.12_Cu_0.12_Mg_0.12_Fe_0.15_Co_0.15_Mn_0.1_Ti_0.1_Sn_0.1_Sb_0.04_O_2_ was reported as a cathode material for SIBs.^[^ ^23^ ^]^ Different from traditional O3‐type cathodes, which suffer from complex phase transitions and poor capacity retention, the HEO exhibited highly reversible O3’–P3 phase transition and outstanding cycling stability. It is suggested that the high entropy effect can help to accommodate local changes and stabilize crystal structure of the materials.^[^ ^23^, ^25^ ^]^ NASICON structure has good compositional flexibility and can incorporate many active transition‐metal (TM) species, such as V, Mn, Cr, Fe, Ti, etc. If the HE concept works in NASICON cathodes, one would expect that increasing the number of TM species would enable multicationic redox reactions and alleviate the degradation of structure. As a proof of concept, here, we develop a novel HE NASICON material Na_3.4_Fe_0.4_Mn_0.4_V_0.4_Cr_0.4_Ti_0.4_(PO_4_)_3_ (HE‐NASICON) following the Boltzmann and Gibbs interpretation of entropy. The structure details of the designed HE materials and the role of each redox active center in the HE‐NASICON are revealed by a series of HE composition design. We find that the structural degradation of NASICON cathodes within a wide operation voltage range can be effectively suppressed by using five TM species with equal molarity, leading to substantial improvements in capacity and rate capability. Specifically, a remarkably high capacity of 161.3 mA h g^−1^ (≈1.5–4.5 V vs Na/Na^+^) with robust structure stability can be enabled by the reversible multi‐Na‐ions intercalation/deintercalation process.

## 2 Results and Discussion

**Figure 1:**
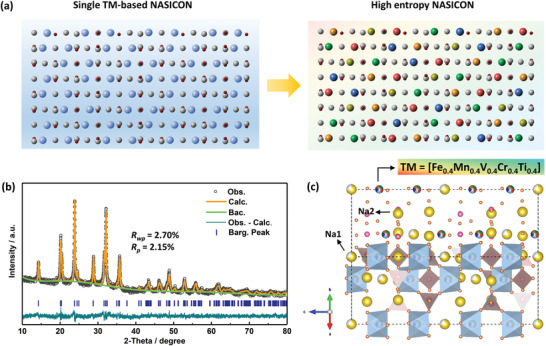
Crystal phase of HE‐NASICON. a) Schematic illustration of the transition‐metals (TMs) distribution in NASICON structure. The gray and red balls represent P and O atoms, respectively. The balls with other colors represent different types of TMs. b) Rietveld refinement profile of the HE‐NASICON material. c) Schematic illustration of the crystal structure of HE‐NASICON.

All HE‐NASICON materials with different concentration of TM species were successfully synthesized using a general sol–gel method on the basis of the molar configuration entropy (Δ*S = −R*(*Σ x_i_ lnx_i_*)_cation‐site_, *x_i_* represents the mole fraction of TM species in the cation sites).^[^ ^17^ ^]^ Compared to the common (low‐entropy) NASICON cathodes with single/double redox centers, the HE‐NASICON material is designed to locate five different TM species (Fe, Mn, V, Ti, Cr) at the cation sites of the crystal lattice with equal atomic concentration, as schematically illustrated in **Figure** **1**a. X‐ray diffraction (XRD) and Rietveld refinement (Figure 1b) reveal that the HE‐NASICON adopts a trigonal crystal phase with space group of *R‐*3c, and lattice parameters were determined to be a = b = 8.7158 Å, c = 21.7979 Å, V = 1434.05 Å^3^ (see more refined parameters in Table S2, Supporting Information). All data confirm the formation of single‐phase NASICON compound with no observable impurity peaks regardless of the complexity of the composition. We used a crystal configuration to understand the detailed structure of the HE‐NASICON. As depicted in Figure 1c, the five TMs (Fe, Mn, V, Ti, Cr) occupy the octahedra site of *12c* with equal molarity, and the TMO_6_ octahedra and PO_4_ tetrahedra are corner‐shared to construct the 3D framework structure. The vibrations of TM‐O bonds in TMO_6_ octahedra and P‐O bonds in PO_4_ tetrahedra were observed by Fourier transform infrared (FT‐IR) spectrum (Figure S1, Supporting Information). Besides, the peaks in the regions of 500–1200 cm^−1^ reveal a typical vibration fingerprint of NASICON structure.^[^ ^26^, ^27^ ^]^

**Figure 2:**
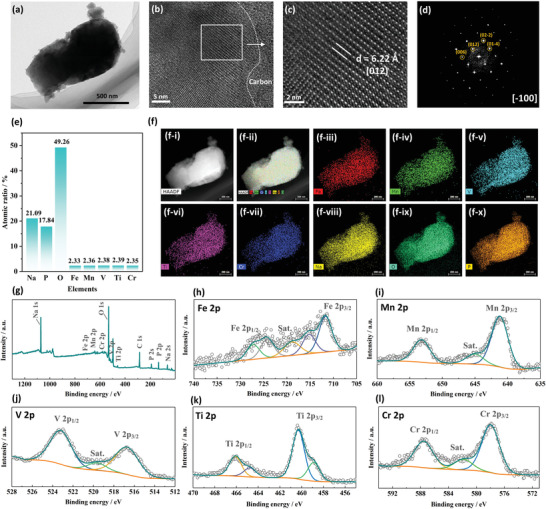
Physicochemical properties of the HE‐NASICON. a) TEM image, b) high‐resolution TEM (HR‐TEM) image, c) enlarged HR‐TEM with clear atomic lattice, and d) the corresponded FFT pattern of the HE‐NASICON material. e) Atomic ratio and f) distributions of Fe, Mn, V, Ti, Cr, Na, O, and P elements in the HE‐NASICON particle. g) XPS survey spectrum of the HE‐NASICON. h) Fe 2p, i) Mn 2p, j) V 2p, k) Ti 2p, and l) Cr 2p core‐level XPS spectra of HE‐NASICON.

Spherical‐aberration‐corrected transmission electron microscope (ACTEM) equipped with energy dispersive spectrometer (EDS) was applied to investigate the detailed structural characteristics and the distribution of the multiple elements in the HE materials (**Figure** **2**a–d). Both TEM (Figure 2a) and scanning electron microscopy (SEM) (Figure S2, Supporting Information) analysis reveal that the as‐synthesized materials display the polyhedral shaped particles with a micrometer‐scale size distribution. A clear carbon/crystal lattice interface can be identified by high‐resolution TEM analysis, demonstrating a typical carbon coating structure (Figure 2b). In addition, thermogravimetry (TG, Figure S3, Supporting Information) reveals that the carbon amount in the HE‐NASICON compound is ≈6.78%, which derives from the pyrolysis of organic species in the raw materials. Further TEM and fast Fourier transform (FFT) analysis of the crystal lattice (Figure 2c,d) reveals that the (006), (012), (02‐2), and (01‐4) facets belong to a typical NASICON crystal, which is consistent with the Rietveld refinement result. The EDS mapping and elemental analysis of a representative particle of the as‐synthesized HE‐NASICON demonstrates equal atomic ratio of the TM species (Figure 2e; and Figure S4, Supporting Information) and uniform distribution of all the elements in the particle (Figure 2f).

Having confirmed the phase purity and crystal structure details of the HE‐NASICON material, we then turn to investigate the valence states of the TM species in the HE‐NASICON material because they were found to be the primary factors in determining the redox process during sodiation/desodiation process. Survey spectrum of the X‐ray photoelectron spectroscopy (XPS) confirms the existence of Na, P, O, Fe, Mn, V, Ti, and Cr in the HE‐NASICON (Figure 2g). The detailed spectra of each TM species are also probed to analyze their chemical states (Figure 2h–l). The characteristic peaks of Fe 2p spectra that located at 711.68 eV (Fe 2p_3/2_) and 724.18 eV (Fe 2p_1/2_) correspond to Fe^2+^ species; while the peaks at 715.18 eV (Fe 2p_3/2_) and 727.28 eV (Fe 2p_1/2_) correspond to Fe^3+^ species and the portion is about 39.4%.^[^ ^28^, ^29^ ^]^ For Mn 2p, the peaks located at 741.28 eV (Mn 2p_3/2_) and 753.28 eV (Mn 2p_1/2_) indicate the 2+ oxidation state of Mn.^[^ ^30^, ^31^ ^]^ The peaks at 516.78 and 523.28 eV in the V 2p spectrum can be indexed to the V^3+^ species.^[^ ^14^, ^32^ ^]^ The Ti also shows mixed states as the Fe does. Specifically, the Ti 2p_3/2_ and Ti 2p_1/2_ peaks at 458.88 and 464.78 eV should be assigned to the Ti^3+^ species, while the peaks at 460.28 and 466.08 eV imply the Ti^4+^ species. The Ti^3+^ is around 33.8%.^[^ ^33^, ^34^ ^]^ For the Cr 2p spectrum, it shows two main peaks at 577.98 eV (Cr 2p_3/2_) and 587.58 eV (Cr 2p_1/2_), respectively, which can be ascribed to the Cr^3+^ species.^[^ ^31^ ^]^ The overall charge state of TM species was calculated to be 5.62, which is very close to the ideal value of 5.60 in the Na_3.4_Fe_0.4_Mn_0.4_V_0.4_Cr_0.4_Ti_0.4_(PO_4_)_3_.

**Figure 3:**
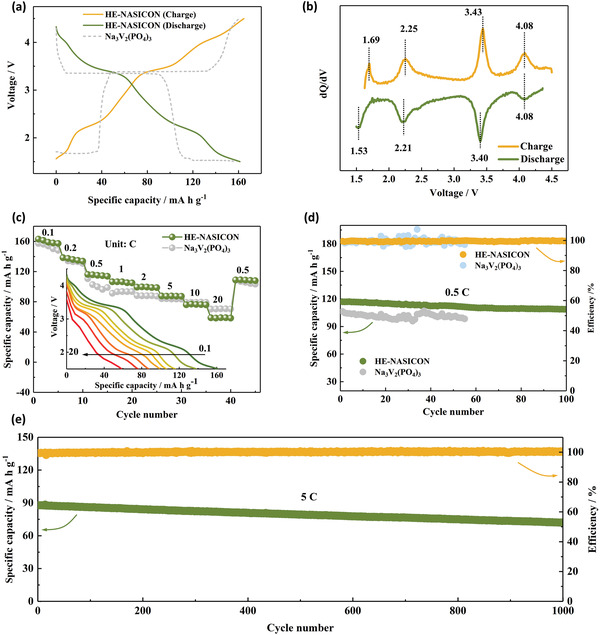
Electrochemical performances of the HE‐NASICON and Na_3_V_2_(PO_4_)_3_ cathode based on multiredox reaction. a) Typical charge/discharge profiles of the cathodes in the full voltage window of 1.5–4.5 V at 0.1 C. b) Corresponded d*Q*/d*V* plot of the HE‐NASICON cathode. c) Rate performance from 0.1 to 20 C. The inset is the corresponded discharge curves of the HE‐NASICON at different rates. d) Cycling performance of the HE‐NASICON and Na_3_V_2_(PO_4_)_3_ at 0.5 C. e) Long cycling stability of the HE‐NASICON at 5 C.

After confirming the successful synthesis of HE‐NASICON, electrochemical performance of the material was then investigated. The HE‐NASICON half‐cells were operated in the voltage range between 1.5 and 4.5 V (vs Na^+^/Na). The typical NASICON Na_3_V_2_(PO_4_)_3_ material with single TM center and multiredox reaction was also studied for comparison. As shown in **Figure** **3**a, the HE‐NASICON can deliver a reversible capacity reaching 161.3 mA h g^−1^ at 0.1 C (1 C = 150 mA g^−–1^). The capacity is higher than that of Na_3_V_2_(PO_4_)_3_, which displays a discharge capacity of 156.5 mA h g^−1^ based on the redox of V^3+^/V^4+^ at 3.4 V and V^2+^/V^3+^ at 1.53 V. From the charge/discharge profiles, it can be seen that redox voltages of the HE‐NASICON are also improved, which is important to improve energy density of the material. The profiles exhibit a multisteps pattern, indicating consecutive redox reactions of multiple TM species in the HE‐NASICON materials.^[^ ^35^ ^]^ Four pairs of redox potentials at 1.69/1.53, 2.25/2.21, 3.41/3.40, and 4.08/4.08 V are found in the d*Q*/d*V* plot. The redox peaks may correspond to the V^2+^/V^3+^, Ti^3+^/Ti^4+^, V^3+^/V^4+^, and V^4+^/V^5+^ redox, respectively. Of note, redox potential of Mn^2+^/Mn^3+^ (≈3.5 V) and Mn^3+^/Mn^4+^ (≈4.0 V) are close to V^3+^/V^4+^ and V^4+^/V^5+^. Therefore, TM species that participate the redox reactions in the HE‐NASICON need further investigation (discussed later). The small gaps between the oxidation and reduction potential of each redox pair indicate highly reversible Na‐ions storage process with good electrode kinetics. As shown in Figure 3c, at the current density of 0.1, 0.2, 0.5, 1, 2, 5, 10, and 20 C, the HE‐NASICON exhibits a discharge capacity of 163.0, 138.3, 116.2, 106.4, 99.4, 87.7, 76.1, and 58.8 mA h g^−1^, respectively. When the current returns to 0.5 C from the high‐rate of 20 C, the discharge capacity can achieve 109 mA h g^−1^, implying good reversibility for flexible Na‐ions storage. Although Na_3_V_2_(PO_4_)_3_ possesses outstanding high‐rate capability above 10 C, the discharge capacity of HE‐NASICON is higher from 0.1 to 5 C. For multi‐Na‐ions storage within a wide voltage window, cyclability is a key issue.^[^ ^16^, ^35^ ^]^ As exhibited in Figure 3d, cycling stability of the Na_3_V_2_(PO_4_)_3_ is less satisfactory, showing greatly fluctuated efficiency. While cycling stability of the HE‐NASICON is significantly enhanced, with a capacity retention of 93.0% after 100 cycles at 0.5 C. Further increasing the current rate to 5 C, a capacity retention of 85.3% after 1000 cycles can still be achieved (Figure 3e). Comparing to some other reported NASICON materials (one/two TM redox center), cycling stability of the HE‐NASICON is also good, with around 0.015% capacity decay of per cycle (Figure S5, Supporting Information).

**Figure 4:**
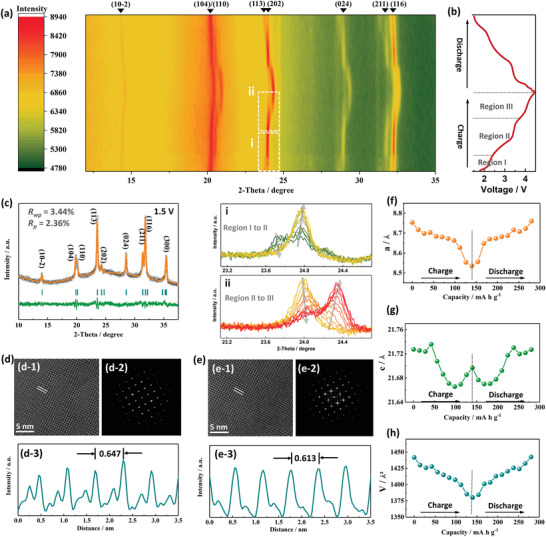
Characterization of the reaction process of HE‐NASICON cathode. a) Contour map of in situ XRD pattern of the HE‐NASICON electrode in the voltage range between 1.5 and 4.5 V (vs Na^+^/Na). The i and ii areas highlight peaks variation in the 2‐theta range of ≈23.1°–24.7°. b) The corresponded charge/discharge curves of the cell operated at current density of 25 mA g^−1^. c) XRD pattern of the electrode at 1.5 V. d) Lattice structure of the electrode at 1.5 V. d‐1) The HR‐TEM and d‐2) the corresponded FFT pattern. d‐3) Integrated pixel intensities collected from e‐1). e) Lattice structure of the electrode at 4.5 V. e‐1) The HR‐TEM and e‐2) the corresponded FFT pattern. e‐3) Integrated pixel intensities collected from i‐1. f) The variation of cell parameters of a, g) c, h) V.

Having confirmed that the high capacity and long‐term cyclic performance arise from the benefits of increasing the TM variety, it remains unclear why the HE‐NASICON can maintain high structural stability than the one/two redox‐centered cathode. Identifying the phase evolution of the cathode is key to understand this type of reinforced behavior in the HE‐NASICON structure. Previous studies using operando XRD have shown that Na‐ions insertion/deinsertion in the Mn‐V binary cathode structure gives rise to large lattice distortion.^[^ ^15^, ^16^ ^]^ In the present study, the effects of TM variety on the crystal lattice during sodiation/desodiation process of the HE‐NASICON cathode were studied using both operando (**Figure** **4**a,b) and regular XRD measurements (Figure 4c), and the diffractions patterns were modeled using full pattern analysis by Rietveld refinement as well as by line profile analysis (Table S3, Supporting Information).

Analysis of the peaks reflection and positions of the full spectra and induvial operando XRD peaks in the voltage range from 1.5 to 4.5 V (vs Na^+^/Na) showed that the diffraction peaks at around 13.9°, 19.8°, 20.2°, 23.6°, and 24.2° refer to (10‐2), (104), (110), (113), and (202) reflection, respectively, and the full desodiation process can be divided into 3 regions (Figure 4b). The peaks variation in the 2‐theta range of ≈23.1°–24.7° has been highlighted (Figure 3a‐i,ii) to present the reaction process. In region I (1.5–2.5 V vs Na^+^/Na), the (113) and (202) reflections can be identified clearly. No significant variation has been observed, indicating a gentle solid‐solution reaction process in this voltage range. As Na‐ions continued to extract and entered from region I to II (2.5–3.7 V vs Na^+^/Na), a clear phase transition has been detected. It can be seen that the intensity of (113) gradually reduced and finally vanished in region II, remaining the left‐shifted (202) peaks with enhanced intensity. Apparently, this diffraction peaks variation is associated with the continuous Na content changes in the cathode host along with the valence states of TM species and local coordination environment.^[^ ^29^, ^43^ ^]^ From region II to region III (3.7–4.5 V vs Na^+^/Na), a clear vanishment of (202) and appearance of a new peak has been observed, indicating a two‐phase transition when entering the high voltage region. Normally, in this high voltage window, unfavorable crystal distortion usually appears in the conventional binary phase (Mn‐V based host), which substantially cause mechanical damage of the cathode material.^[^ ^15^, ^16^ ^]^ Interestingly, an iso‐symmetric reaction pattern can be evidenced with the three distinct regions, corresponding to the operando XRD peak reversibility when fully discharged. The peaks shifting and intensity changes should be only ascribed to lattice expanding/shrinking and slight local environmental rearrangement during sodium‐ion extraction/insertion.^[^ ^36^ ^]^ Compared to the binary phase, this reversible sodiation/desodiation process occurs because the synchronized effects of the multiredox centers of the HE‐NASICON prevents the irreversible lattice distortion in high voltage range. This hypothesis is further accessed by investigating the crystal phase of the HE‐NASICON electrode at 4.5 V (Figure S6, details of the Rietveld refinement results are listed in Table S4, Supporting Information). The fully charged electrode can also be indexed to a trigonal *R‐*3c phase, which is the same as the electrode at 1.5 V (Figure 4c). Such a reaction mechanism is also similar to that of LiFePO_4_, which results in FePO_4_ via a two‐phase reaction, while the two endmembers show the same space group (*Pnma*), and it is suggested to benefit stability of the electrode because of their relatively small changes in lattice structure compared to the materials with endmembers of different crystal phases.^[^ ^37^, ^38^ ^]^

To further study the microstructure integrity between sodiation and desodiation process, ACTEM/FFT analysis of the fresh electrode before charging (Figure 4d), fully charged (Figure 4e), and discharged electrodes (Figure S7, Supporting Information) were conducted. FFT patterns of the electrodes can be identified to a trigonal phase along zone‐axis of [42–1]. A lattice spacing of 0.647 and 0.613 nm is measured for the electrodes at 1.5 and 4.5 V, respectively. The decreased spacing confirms the shrinkage of the electrode lattice during desodiation process. When the electrode was fully discharged from 4.5 to 1.5 V, the lattice spacing returned to 0.644 nm (Figure S7, Supporting Information), confirming reversible lattice variation in the stable HE‐NASIOCN crystal structure. As shown in Figure 4f–h, lattice parameter changes of the HE‐NASICON during the reaction are reversible.

**Figure 5:**
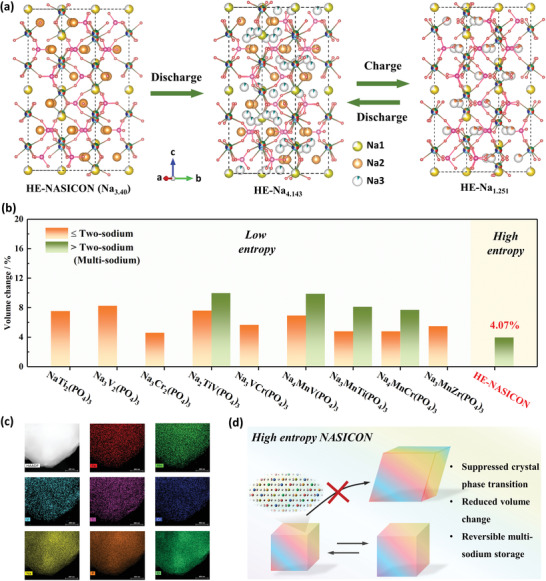
Reaction mechanism of the HE‐NASICON. a) Schematic illustration of the crystal structure evolution of HE‐NASICON during the multi‐sodium storage reaction.b) Comparison of some typical NASICON materials that reported in terms of volume changes when conducing two‐sodium and/or multisodium storage reactions.^[^ ^11^, ^16^, ^39^, ^40^, ^41^, ^42^, ^43^, ^44^, ^45^, ^46^ ^]^ c) The elemental mapping images of the HE‐NASICON electrode after 1000 cycles at 5 C. d) Schematic illustration of the reaction features of the high entropy NASICON material.

On the basis of these operando measurements and microscale observation, the reaction mechanism of the multi‐Na‐ions storage process in the HE‐NASICON was plotted in **Figure** **5**a. The pristine HE‐NASICON, which contains 3.4 Na, was initially discharged to obtain a Na‐rich phase Na_3.4+_ *_x_* (Fe_0.4_Mn_0.4_V_0.4_Ti_0.4_Cr_0.4_)(PO_4_)_3_ (HEN‐Na_3.4+_ *_x_*). The refined *x* value is determined to 0.743 for the material at 1.5 V (Table S3, Supporting Information). In the pristine HE‐NASICON structure, Na‐ions locate at Na1 and Na2 sites. The extra inserted sodium‐ions in HE‐Na_4.143_ partially enters into the Na1 stie, increasing the occupancy factor of Na1 from 0.539 to 0.705. While other inserted sodium‐ions settle in a new Na3 site (36*f*). It has been reported that the Coulombic repulsion, which is caused by simultaneous occupation of neighboring Na1 and Na2 sites, would lead Na ions to Na3 site.^[^ ^39^ ^]^ The fully charged Na‐deficient phase is determined to be Na_1.251_(Fe_0.4_Mn_0.4_V_0.4_Ti_0.4_Cr_0.4_)(PO_4_)_3_ (HEN‐Na_1.251_), where the Na^+^ ions at Na3 site are fully extracted (Table S4, Supporting Information). The number of Na ions participate in energy storage is then calculated to be 2.892, verifying a multi‐Na‐ions storage reaction as anticipated. The quantitative difference of the overall volume change of the NASICON materials,^[^ ^11^, ^16^, ^39^, ^40^, ^41^, ^42^, ^43^, ^44^, ^45^, ^46^ ^]^ summarized in Figure 5b, is not well recognized in the previous reports. In fact, mechanistic variations of the electrodes have been suggested as major cause for phase inhomogeneity during charge/discharge process of the common binary‐phase NASICON materials, such as Na_4_MnV(PO_4_)_3_, Na_3_MnTi(PO_4_)_3_, and Na_4_MnCr(PO_4_)_3_, etc. Note that, it is only 4.07% in the HE‐NASICON material.

To test and validate the mechanistic postulates summarized above, we have carried out the long‐term test at high current for the HE‐NASICON material. After 1000 cycles at 5 C, the crystal phase of the HE‐NASICON material maintains consistently (Figure S8, Supporting Information) as demonstrated by the XRD measurement. The robust microstructure of carbon coated trigonal NASICON lattice after long cycling was also evidenced by the HRTEM characterization (Figure S9, Supporting Information). Furthermore, EDS shows that the atomic ratio of the 3d‐transition‐metals is consistent with the stoichiometric of the HE‐NASICON (Figure S10, Supporting Information), and all the elemental components still uniformly distributed in the material (Figure 5c), indicating no phase segregation after cycling.

The high entropy strategy is suggested to present multifunctions to improve the electrochemical properties of NASICON material, as illustrated in Figure 5d. The HE effect can help increase the solid solubility of different elements in one phase, and strain effect in NASICON materials, which is caused by the rich accommodated atoms with different sizes, leading to an intense lattice strain field that suppresses the large phase transition and lattice variation.^[^ ^19^ ^]^ As a result, unfavorable crystal phase transition is suppressed in the high entropy material even when it was charged to the high voltage of 4.5 V.

**Figure 6:**
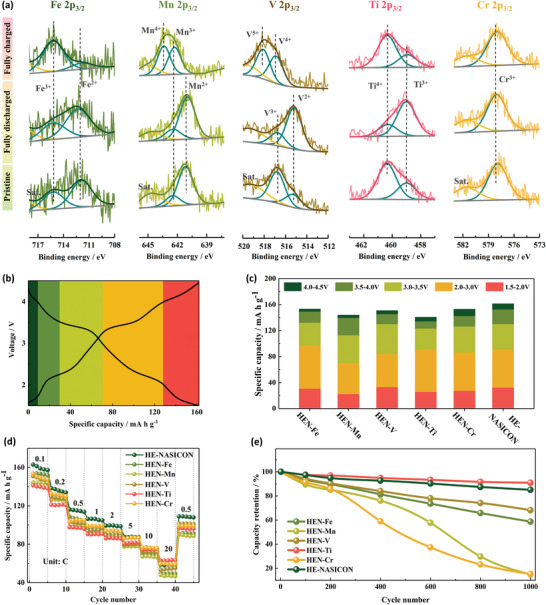
Impact of the various transition‐metals. a) XPS of the Fe 2p_3/2_, Mn 2p_3/2_, V 2p_3/2_, Ti 2p_3/2_, Cr 2p_3/2_ that collected from the pristine electrode, fully discharged electrode (1.5 V), and fully charged electrode (4.5 V) of HE‐NASICON. b) Demonstration of the colors highlighted capacity contribution of voltage regions of ≈1.5–2.0 V (dark green), ≈2.0–3.0 V (green), ≈3.03.5 V (light green), ≈3.5–4.0 V (yellow), and ≈4.0–4.5 V (red) in HE‐NASICON. c) Capacity contribution of the various high entropy NASICON materials at 0.1 C. d) Rate performance and e) cycling stability of the high entropy NASICON materials at 5 C.

Considering the geometric and crystallographic characteristics of the HE‐NASICON material, the following measurements were conducted to elucidate the origin of the highly reversible multi‐Na‐ions storage, together with its linkage with the role of each TM species in the HE structure. Given the composition‐dependent HE‐NASICON structure, it is attempting to connect the multi‐Na‐ions storage to a kinetic‐controlled process that becomes faster with diverse TM‐based redox centers. To access this hypothesis, the specific valence states of each increased TM species in the HE‐NASICON structure need to be understood in the context of homogeneous composition distribution before and after charging process. The valence states of the TM species in the pristine, fully discharged and charged electrodes were analyzed by the ex situ XPS to determine the electrochemically active species during the multi‐Na‐ions storage reaction (**Figure** **6**a). For Fe 2p_3/2_ and Mn 2p_3/2_, their profiles present no significant changes between pristine and fully discharged electrodes, indicating that Fe and Mn do not participate redox reaction during the initial discharge process. While when the electrode was fully charged, the intensity of Fe^2+^ (711.75 eV) decreases and Fe^3+^ (715.21 eV) increases obviously, manifesting that the Fe^2+^/Fe^3+^ redox reaction was activated in the charge process of the HE‐NASICON. The Mn 2p_3/2_ was found to shift to higher binding energy and two main peaks at 642.33 eV (Mn^3+^) and 643.38 eV (Mn^4+^) appeared when fully charged, indicating that the Mn^2+^ was oxidated to a Mn^3+^/Mn^4+^ mixed state.^[^ ^47^ ^]^ Different from Fe and Mn, V and Ti are active during the initial discharge process, as evidenced by the clear intensity increase of V^2+^ (515.31 eV) and Ti^3+^ (458.98 eV) species in V 2p_3/2_ and Ti 2p_3/2_ peaks, respectively.^[^ ^48^, ^49^ ^]^ The Ti^3+^ was found recover back to Ti^4+^ (460.38 eV) in the fully charged sample, while the V^2+^ was oxidated to V^5+^ (518.11 eV) and V^4+^ (516.99 eV), implying that the V undergoes continuous redox reactions.^[^ ^36^, ^50^ ^]^ Cr 2p_3/2_ is the only TM species that almost has no changes during the whole reaction. The XPS results verify the multiactive TM redox centers and multiredox reactions in the HE‐NASICON material, which is in accordance with the multivoltage platforms of the charge/discharge profiles.

For quantitative confirmation of our multi‐Na‐ions storage based on the HE‐NASICON structure, we designed other five compositions with doubled concentration of Fe, Mn, V, Ti, and Cr (noted as HEN‐Fe, HEN‐Mn, HEN‐V, HEN‐Ti, HEN‐Cr, respectively). All the samples showed the same crystal phase as the HE‐NASICON (Figure S11, Supporting Information). EDS mapping demonstrates that the atomic ratios of the elements are close to their theoretical values and the elements are homogeneously distributed, indicating successful synthesis of the diverse HE materials (Figures S12–S16, Supporting Information). Carbon content of the materials were determined by TG (Figure S17, Supporting Information) and were ruled out when counting their sodium storage capacities. Galvanostatic charge/discharge profiles of the samples at 0.1 C are presented in Figure S18 (Supporting Information). The discharge capacity of HEN‐Fe, HEN‐Mn, HEN‐V, HEN‐Ti, and HEN‐Cr is 153.2, 144.0, 150.8, 140.7, and 153.0 mA h g^−1^, respectively. All the discharge curves show multiple terraces. To analyze capacity contributions in different voltage regions, the profiles are divided into 5 regions highlighted with different colors, as demonstrated in Figure 6b. Larger capacity contributions in the high voltage regions of ≈3.5–4.0 and ≈4.0–4.5 V are found in HEN‐Mn and HEN‐Cr, indicating that Mn and Cr are beneficial to achieve high voltage properties of the material (Figure 6c). Rate performance and cycling capability of the materials are exhibited in Figure 6d; and Figure S19 (Supporting Information). For HEN‐Fe, although it has relatively large capacity at 0.1 C, its rate performance is poor, with only 49.9 mA h g^−1^ at 20 C. The HEN‐Mn shows the worst rate capability, which is probably because of the relatively sluggish electrode kinetics as many Mn‐based materials reported.^[^ ^6^, ^51^ ^]^ In the contrast, the HEN‐Ti possess the highest capacity of 62.81 mA h g^−1^ at 20 C, regardless the relatively low capacity at 0.1 C. Besides, HEN‐Ti also demonstrates the best cycling performance, with a remarkable capacity retention of 90.92% after 1000 cycles at 5 C (Figure 6e). While the capacity of HEN‐Cr decays very fast. Therefore, the TM species in the HEN series show different features affecting the materials in terms of capacity, voltage, rate capability, and cycling stability. Roughly, Fe shows a high redox activity to help contribute a large capacity; Mn can lead to an increased voltage due to the redox of Mn^3+^/Mn^4+^; V possesses a multi‐redox feature to ensure the high capacity of the cathodes; Cr is key to enhance the capacity in the high voltage regions and rate performance; Ti is of great significance to increase cycling stability. It is the synergistic effect of TM species with suitable molarity and HE composition finally leads to the high‐performance HE‐NASICON material.

## 3 Conclusion

A high‐entropy NASICON structure Na_3.4_Fe_0.4_Mn_0.4_V_0.4_Cr_0.4_Ti_0.4_(PO_4_)_3_ material was successfully synthesized through a facile sol–gel method. The HE‐NASICON is found to possess multiredox reactions due to the diverse TMs species and overcome the stability and reversibility issues of general polyanion type cathode materials. In situ XRD analysis shows that crystal phase of the HE‐NASICON during the multi‐Na storage reaction is stable, and the volume change is small. It is suggested that the high entropy effect can help to stabilize the host framework of NASICON structure, thus leading to the enhanced cyclability. By comparing a group of HE‐NASICON cathodes containing different contents of TM species, we further demonstrate the advanced electrochemical performance of HE‐NASICON also derives from the synergistic effect of the various transition metals with equal molarity. The high entropy strategy on NASICON opens a new opportunity to design advanced polyanion compounds for SIBs.

## Conflict of Interest

The authors declare no conflict of interest.

## Supporting information

Supporting Information

## Data Availability

Research data are not shared.
