# Supplementary material for: Stabilization of Multicationic Redox Chemistry in Polyanionic Cathode by Increasing Entropy
Source: Adv Sci (Weinh). 2022 Jul 1;9(25):2202082. doi: 10.1002/advs.202202082 (PMC9443449; doi:10.1002/advs.202202082)
Supplement: Supplementary file 1 — Supporting Information [file ADVS-9-2202082-s001.pdf]

ADVANCED SCIENCE
Open Access

Supporting Information

for *Adv. Sci.*, DOI 10.1002/advs.202202082

Stabilization of Multicationic Redox Chemistry in Polyanionic Cathode by Increasing Entropy

Huangxu Li, Ming Xu, Huiwu Long, Jingqiang Zheng, Liuyun Zhang, Shihao Li, Chaohong Guan, Yanqing Lai* and Zhian Zhang**

Supplementary material

Stabilization of Multi-Cationic Redox Chemistry in Polyanionic Cathode by Increasing Entropy

Huangxu Li,[a,b] Ming Xu,[c,*] Huiwu Long,[b] Jingqiang Zheng,[a] Liuyun Zhang,[a] Shihao Li,[a] Chaohong Guan,[d] Yanqing Lai,[a,*] Zhian Zhang [a,*]

[a] School of Metallurgy and Environment, Engineering Research Center of the Ministry of Education for Advanced Battery Materials, Hunan Provincial Key Laboratory of Nonferrous Value-Added Metallurgy Central South University, Changsha 410083, P. R. China

[b] Department of Chemistry, City University of Hong Kong, Kowloon, Hong Kong 999077, P. R. China

[c] School of Chemistry, Xi'an Jiaotong University, Xi'an 710049, P.R. China

[d] University of Michigan−Shanghai Jiao Tong University Joint Institute, Shanghai Jiao Tong University, Shanghai 200240, P. R. China

Corresponding email:

xuming@xjtu.edu.cn

laiyanqing@csu.edu.cn

zhangzhian@csu.edu.cn

Experimental section

Synthesis of HE-NASICON and other high-entropy NASICON materials with different atomic ratio. The HE-NASICON was synthesized by a typical sol-gel method. All chemical reagents were used without further purification. Iron nitrate nonahydrate, manganese acetate, ammonium metavanadate, titanium (IV) isopropoxide, and chromium(III) nitrate nonahydrate were used as the Fe, Mn, V, Ti, Cr sources, respectively. Firstly, stoichiometric amount of anhydrous citric acid and ammonium metavanadate are dissolved in 20 mL deionized water, forming solution A. Then, sodium carbonate and the Fe, Mn, Cr sources are added in another 40 mL deionized water, forming solution B. A was then mixed with B at 80 °C and kept constant stirring for 30 min. After that, ammonium biphosphate were added into the mixed solution. When the ammonium biphosphate was fully dissolved, appropriate amount of titanium (IV) isopropoxide that dissolved in 20 mL absolute ethanol was finally added into the mixed solution drop by drop. The final solution kept constant stirring at 80 °C until a gel was formed. The gel was dried at 120 °C in an oven. Finally, the porous precursor was calcinated at 700 °C for 12 h under Ar to obtain the HE-NASICON material. Other high-entropy NASICON materials with different atomic ratio were also synthesized through the same sol-gel route. The HEN-Fe, HEN-Mn, HEN-V, HEN-Ti, HEN-Cr represents the material with doubled atomic concentration of Fe, Mn, V, Ti, Cr, respectively. The amounts of reagents used to synthesize the materials are listed in Table S1.

Materials characterization. X-ray diffraction (XRD) data and were collected from 10° to 80° in PANalytical Empyrean 2. The XRD refinements were conducted by GSAS software. Morphology and composition of the materials was disclosed by field-emission scanning electron microscopy (SEM, JSM-IT300LA) equipped with energy-dispersive X-ray spectroscopy (EDS). Micro-structure of materials was further studied by spherical-aberration-corrected transmission electron microscopy (ACTEM, Titan G2 60-300). Electron diffraction images were got by Fast Fourier Transform (FFT). Valence states of Fe, Mn, V, Ti and Cr were determined by X-ray photoelectron spectroscopy (XPS, ESCALAB 250Xi). Fourier transform infrared spectroscopy (FT-IR, Thermo Scientific Nicolet iS50) was utilized to characterize the chemical bonds. Carbon content of the materials were determined by thermogravimetric (TG) analysis from room temperature to 650 °C with a heating rate of 10 °C min^{-1} under air atmosphere.

Electrochemical measurements. For the fabrication of electrodes for electrochemical performance tests, the active materials, carbon black and polyvinylidene fluoride (PVDF) with a weight ratio of 7: 2: 1 were mixed in appropriate amount of 1-Methyl-2-pyrrolidinone (NMP) to make a slurry. The slurry was costed on Al foil and dried at 80 °C. Loading mass of active materials was in the range of 1.2~1.4 mg cm^{-2}. Na metal and glass fiber filters (Whatman) were used as counter electrode and separator, respectively. 1M $NaClO_4$ dissolving in PC solution with 5 vol% addition of

fluoroethylene carbonate (FEC) was used as the electrolyte. Electrochemical performance of the cells was carried out on a Land battery testing system (CT2001A) within the voltage range of 4.5-1.5 V (vs. Na/Na^+). The amounts of carbon are dismissed when accounting the capacity of the cathodes.

Table S1. The amounts of reagents used to synthesize the diverse materials

Material	Sodium carbonate	Iron nitrate nonahydrate	Manganese acetate	Ammonium metavanadate	Titanium isopropoxide	Chromium nitrate nonahydrate	Ammonium biphosphate
HE-NASICON	5.10 mmol	1.20 mmol	1.20 mmol	1.20 mmol	1.20 mmol	1.20 mmol	9.00 mmol
HEN-Fe	5.50 mmol	2.00mmol	1.00 mmol	1.00 mmol	1.00 mmol	1.00 mmol	9.00 mmol
HEN-Mn	5.50 mmol	1.00 mmol	2.00 mmol	1.00 mmol	1.00 mmol	1.00 mmol	9.00 mmol
HEN-V	5.00 mmol	1.00 mmol	1.00 mmol	2.00 mmol	1.00 mmol	1.00 mmol	9.00 mmol
HEN-Ti	4.55 mmol	1.00 mmol	1.00 mmol	1.00 mmol	2.00 mmol	1.00 mmol	9.00 mmol
HEN-Cr	5.00 mmol	1.00 mmol	1.00 mmol	1.00 mmol	1.00 mmol	2.00 mmol	9.00 mmol

Table S2. Refined structure information of the pristine HE-NASICON.

Crystal phase: trigonal, R-3c (S.G.); a=8.7158Å, c=21.7979 Å, V=1434.05 $Å^3$						
Atom	x	y	z	Occ.	Uiso.	Wyckoff. Position
O1	0.00982	0.20866	0.192	1	0.00006	36f
O2	0.1863	0.1721	0.0852	1	0.00006	36f
P	0.31251	0	0.25	1	0.00006	18e
Na1	0	0	0	0.539	0.009	6b
Na2	0.58446	0	0.25	0.970	0.05585	18e
Mn	0	0	0.14901	0.2	0.00006	12c
Fe	0	0	0.14901	0.2	0.00006	12c
V	0	0	0.14901	0.2	0.00006	12c
Ti	0	0	0.14901	0.2	0.00006	12c
Cr	0	0	0.14901	0.2	0.00006	12c

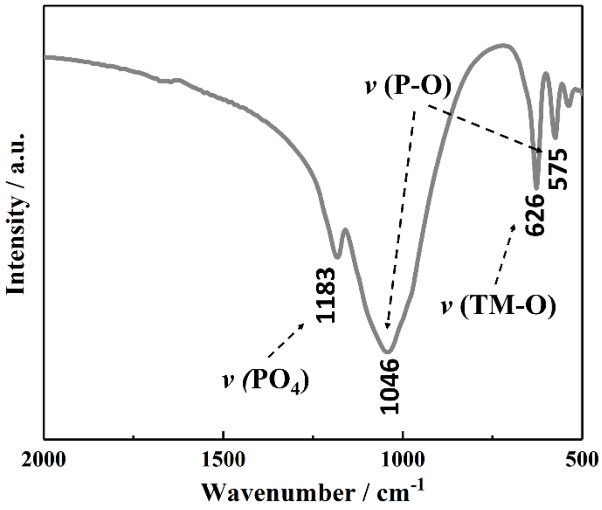

Figure S1. The Fourier transform infrared (FT-IR) spectrum of the HE-NASICON material. The peak at 626 cm^{-1} can be assigned to the vibration of TM-O bonds in TMO_6 octahedra, and the peak at 575 cm^{-1} and 1046 cm^{-1} indicates vibration of P-O bonds. The infrared band at 1183 cm^{-1} can be attributed to stretching of PO_4 tetrahedra. [1, 2]

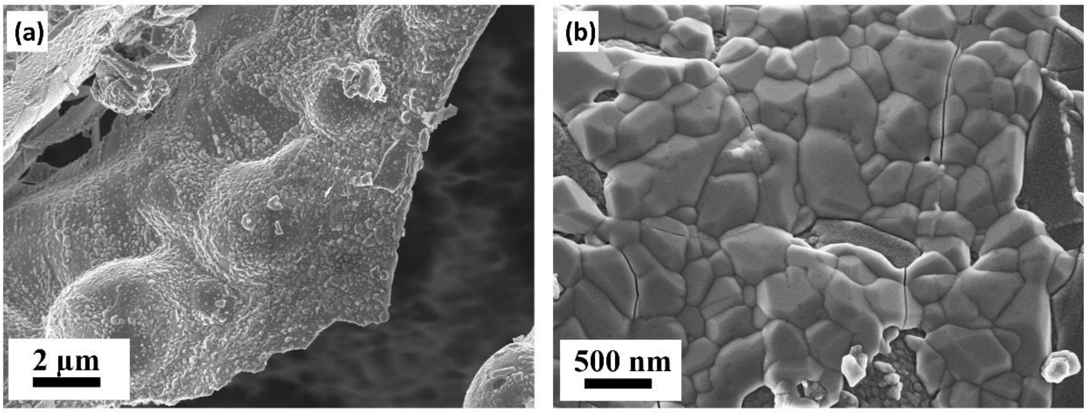

Figure S2. (a) SEM image and (b) high magnification SEM image of the as-synthesized HE-NASICON material.

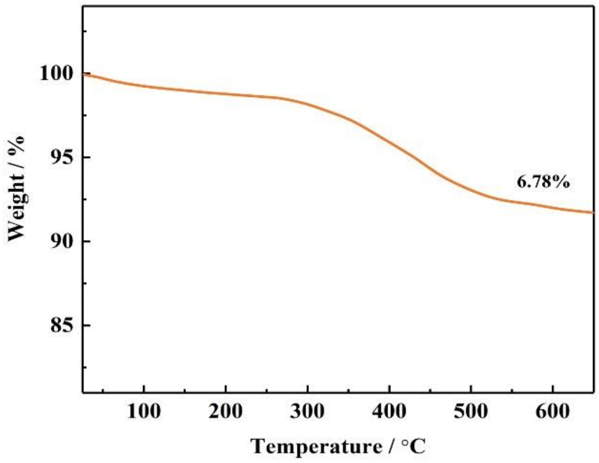

Figure S3. Thermogravimetric analysis of the HE-NASICON under air.

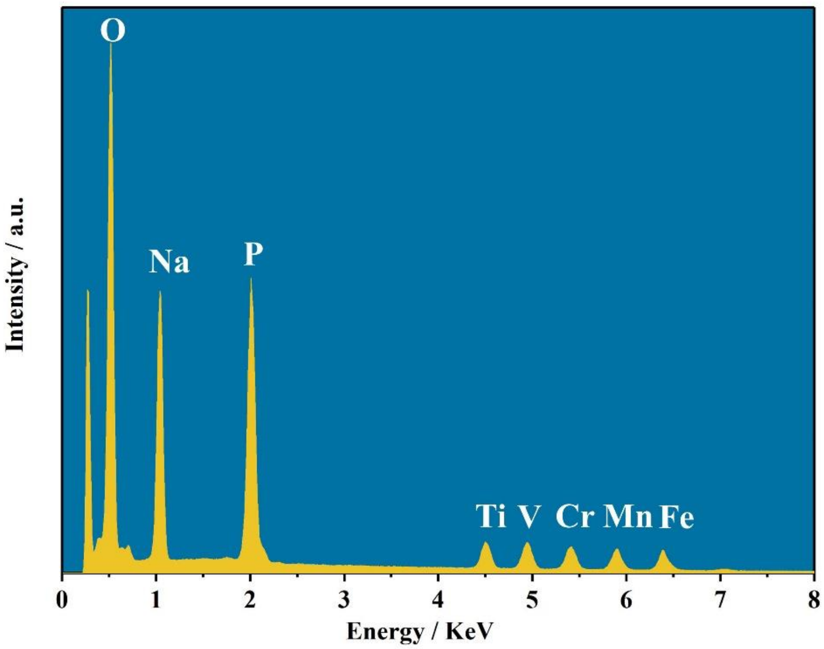

Figure S4. EDS of the HE-NASICON.

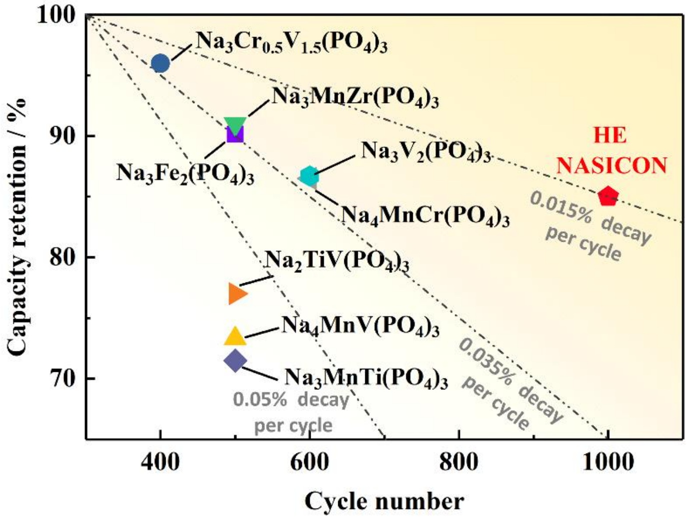

Figure S5. Cycling stability of the HE-NASICON with some other typical NASICON materials that previously reported. For a fair comparison, the data adopted are materials with simple carbon compositing as the HE-NASICON does. [3-10]

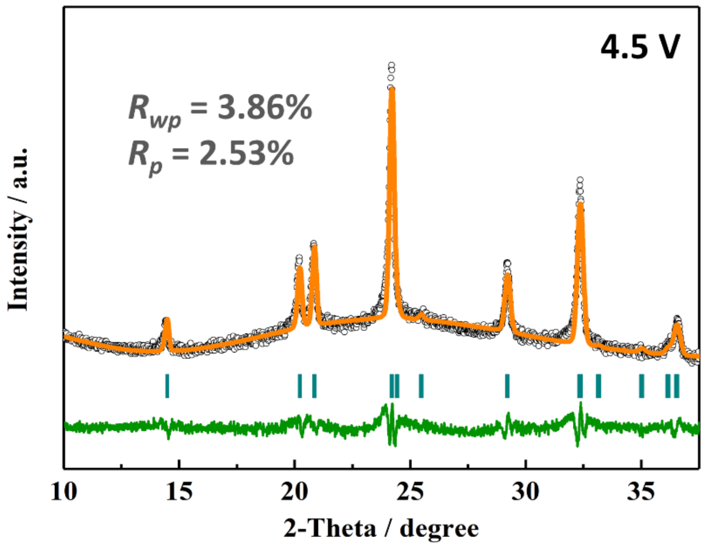

Figure S6. XRD pattern of the electrode charged at 4.5 V.

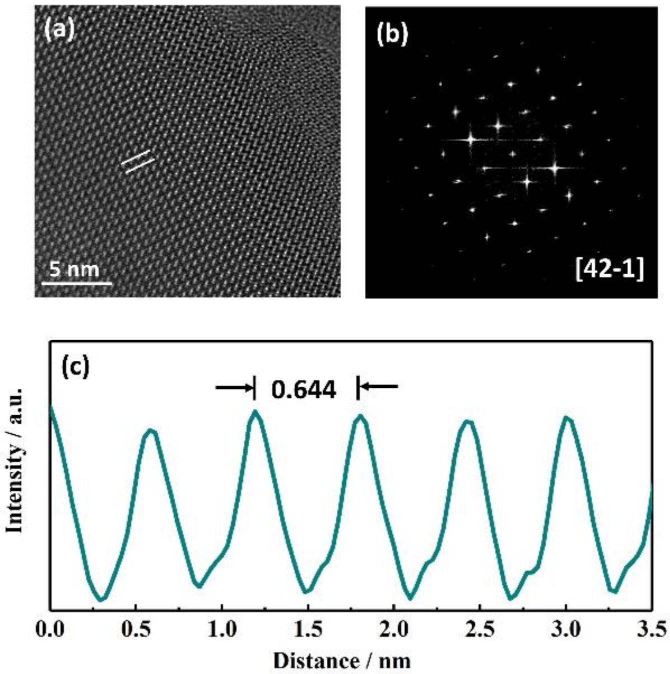

Figure S7. Lattice structure of the electrode charged to 4.5 V and then discharged to 1.5 V. (a) The ACTEM and (b) the corresponded FFT pattern. (c) Integrated pixel intensities collected from a.

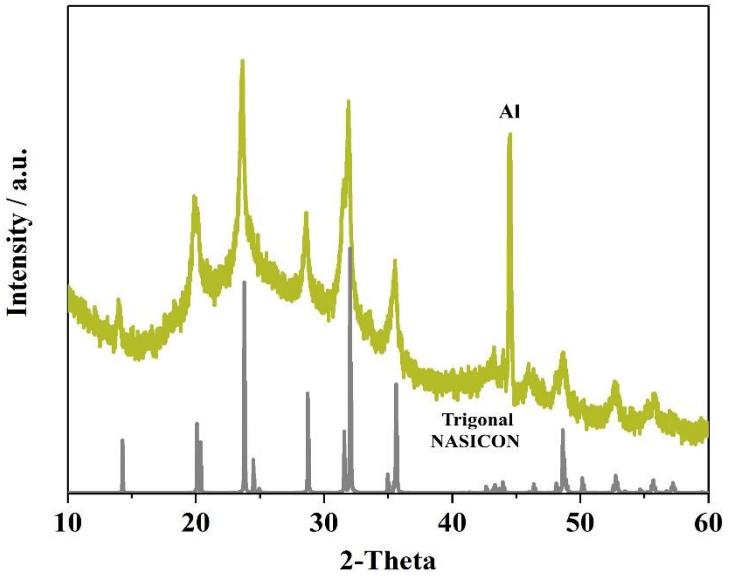

Figure S8. XRD of the HE-NASICON electrode after 1000 cycles at 5 C.

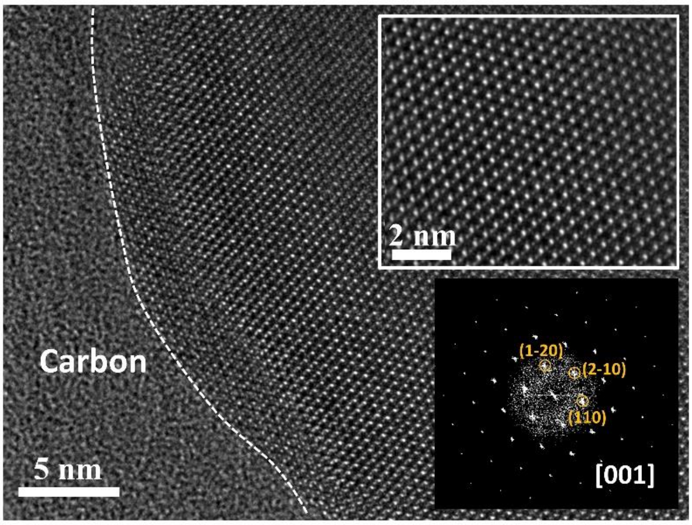

Figure S9. HR-TEM images of the HE-NASICON electrode after 1000 cycles at 5 C. Insets are the enlarged image of the crystal lattice and the corresponded FFT pattern.

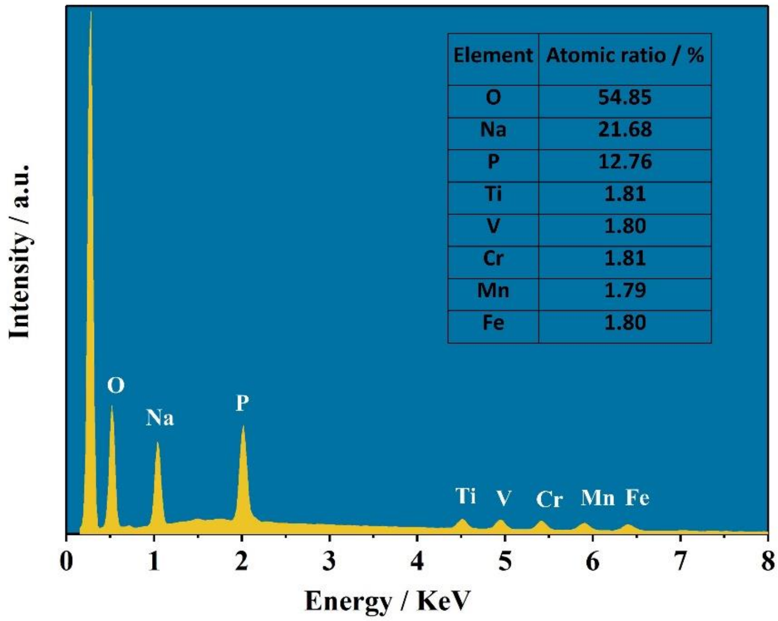

Figure S10. EDS of the HE-NASICON electrode after 1000 cycles at 5 C.

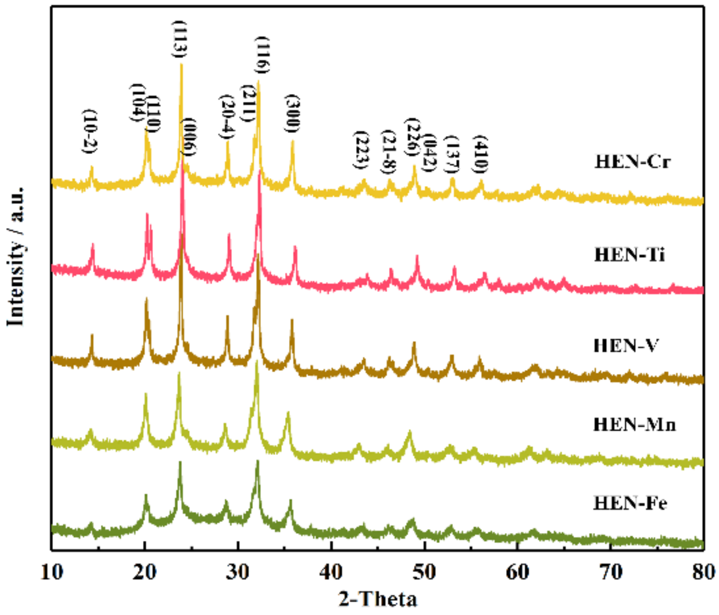

Figure S11. XRD of the HEN-Fe, HEN-Mn, HEN-V, HEN-Ti, and HEN-Cr.

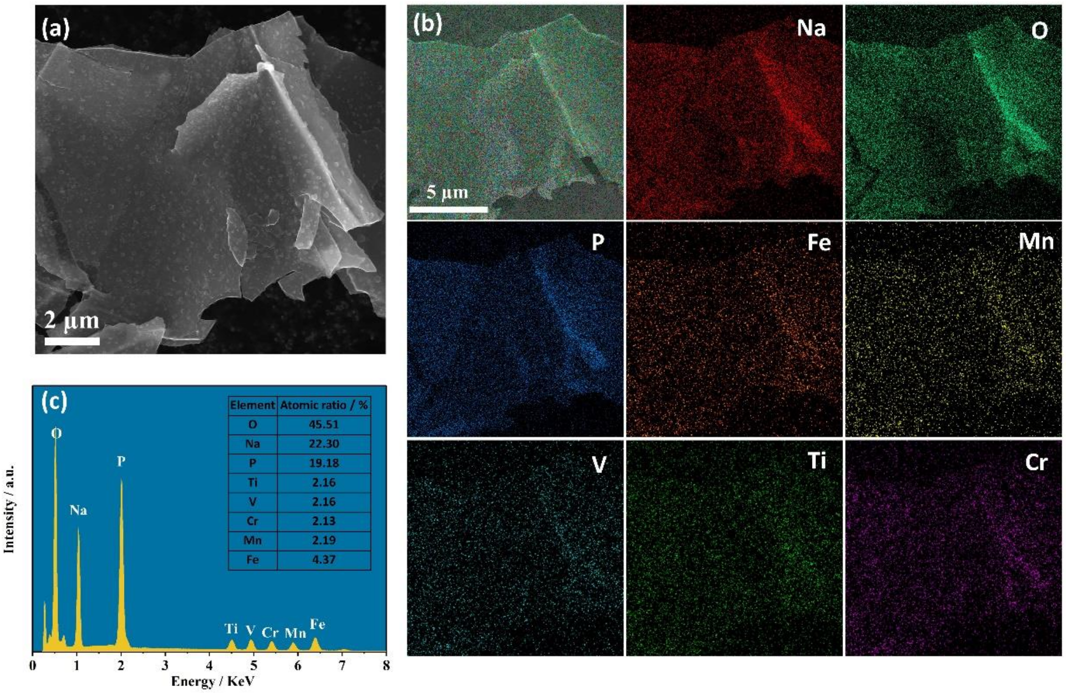

Figure S12. (a) SEM, (b) the elemental mapping, and (c) EDS of the HEN-Fe material.

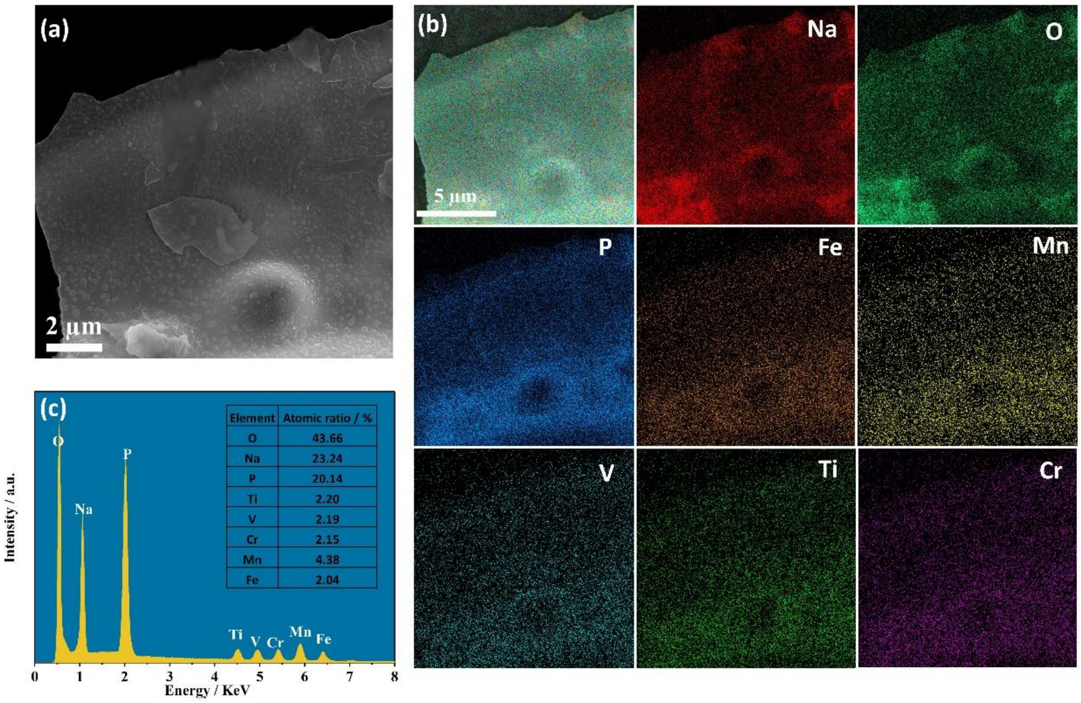

Figure S13. (a) SEM, (b) the elemental mapping, and (c) EDS of the HEN-Mn material.

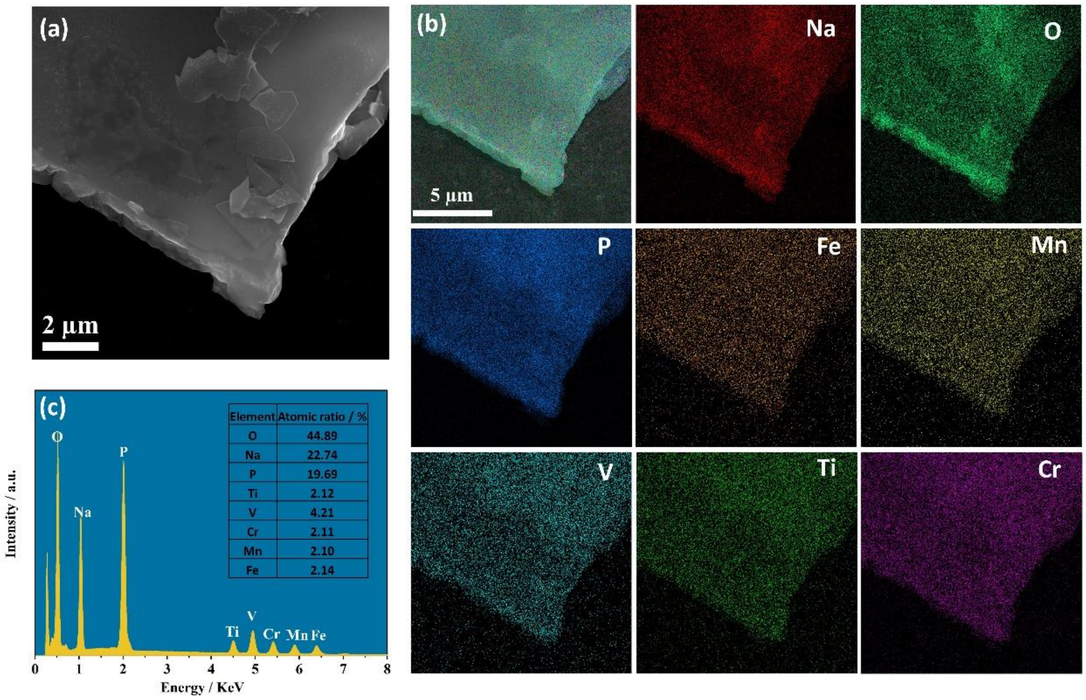

Figure S14. (a) SEM, (b) the elemental mapping, and (c) EDS of the HEN-V material.

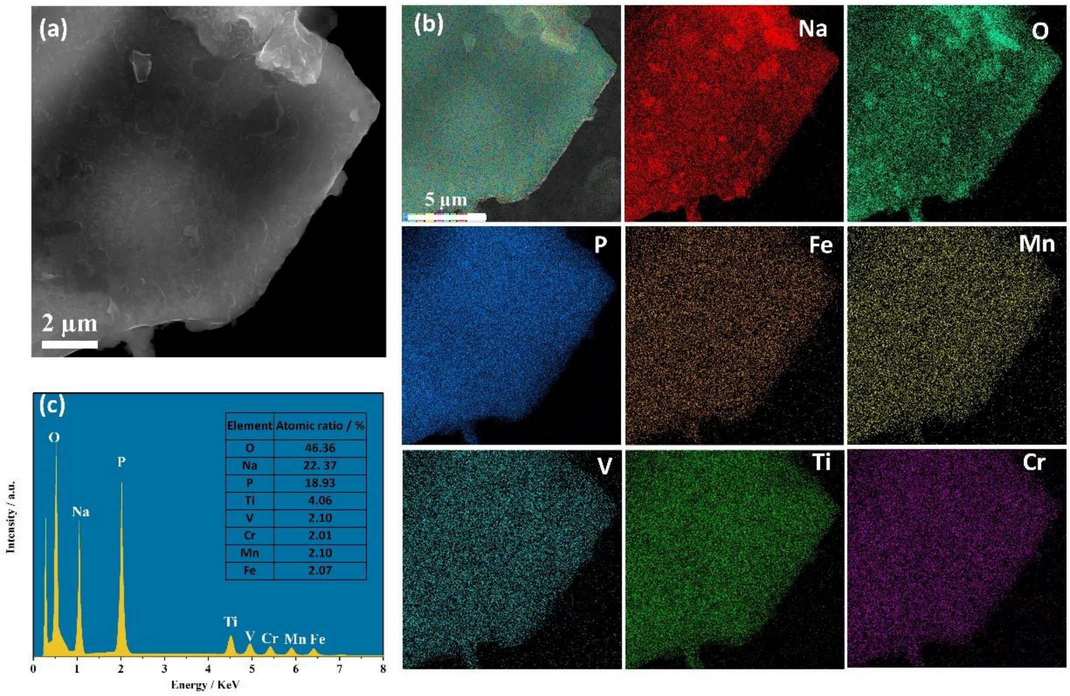

Figure S15. (a) SEM, (b) the elemental mapping, and (c) EDS of the HEN-Ti material.

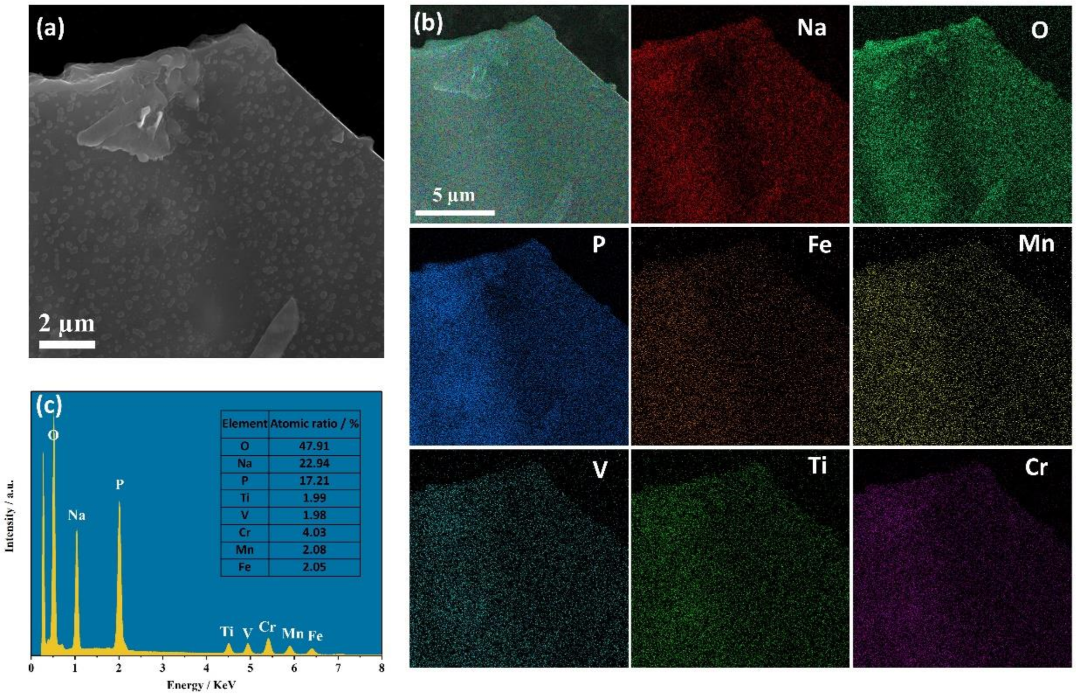

Figure S16. (a) SEM, (b) the elemental mapping, and (c) EDS of the HEN-Cr material.

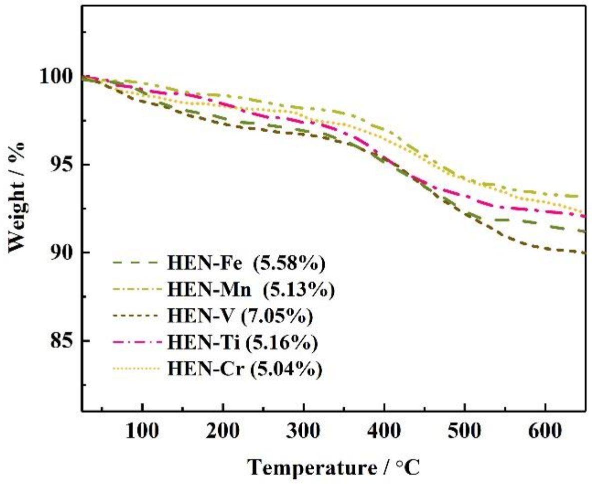

Figure S17. TG curves and carbon contents of the (a) HEN-Fe, (b) HEN-Mn, (c) HEN-V, (d) HEN-Ti, and (e) HEN-Cr.

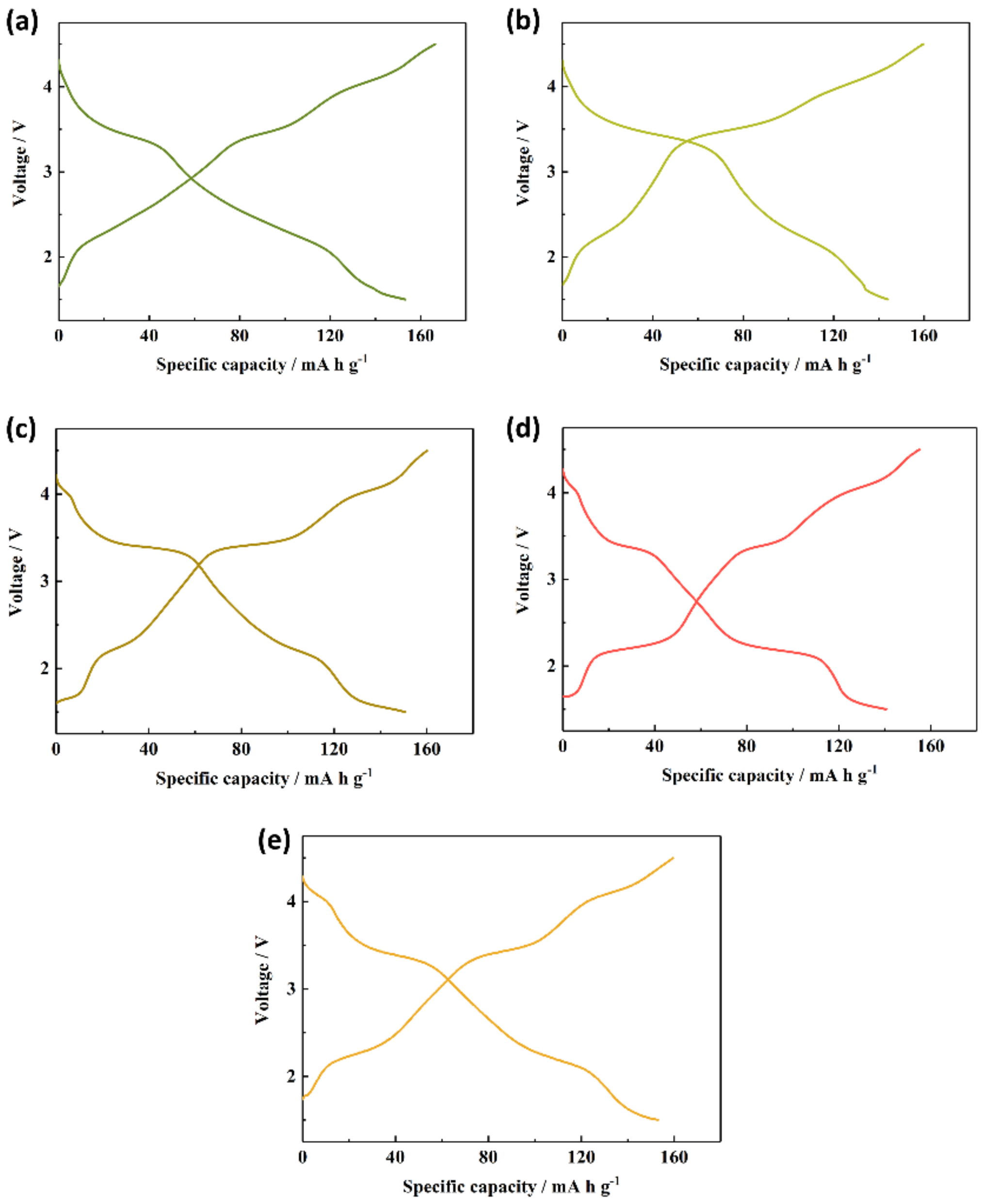

Figure S18. Charge/discharge curves of the (a) HEN-Fe, (b) HEN-Mn, (c) HEN-V, (d) HEN-Ti, and (e) HEN-Cr at 0.1 C.

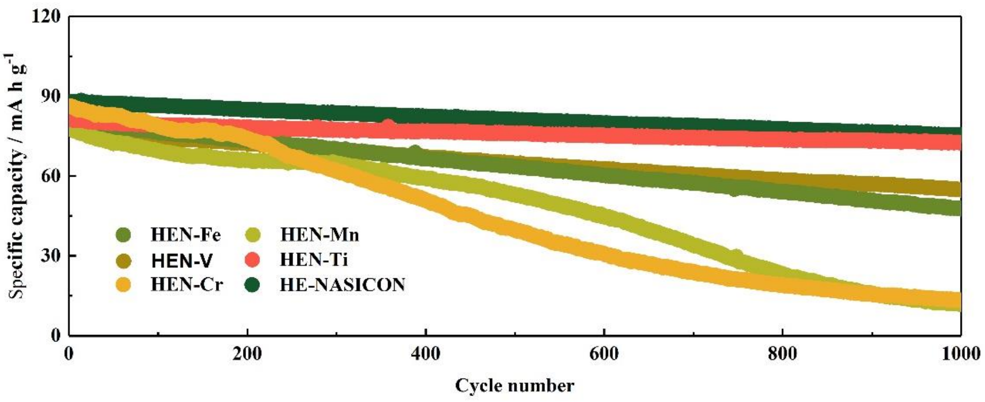

Figure S19. Cycling performance of the diverse high entropy materials at 5 C.

Table S3. Refined structure information of the HE-NASICON electrode at 1.5 V.

Crystal phase: trigonal, R-3c (S.G.); a=8.731755 Å, c=21.823671 Å, V=1438.02 Å^3						
Atom	x	y	z	Occ.	Uiso.	Wyckoff. Position
O1	0.0469	0.2297	0.1975	1	0.07384	36f
O2	0.1754	0.1529	0.0806	1	0.07081	36f
P	0.3008	0	0.2500	1	0.01438	18e
Na1	0	0	0	0.705	0.21503	6b
Na2	0.6273	0	0.25	0.972	0.05585	18e
Na3	0.4703	0.4047	0.3743	0.087	0.22293	36f
Mn	0	0	0.1689	0.2	0.00926	12c
Fe	0	0	0.1689	0.2	0.00926	12c
V	0	0	0.1689	0.2	0.00926	12c
Ti	0	0	0.1689	0.2	0.00926	12c
Cr	0	0	0.1689	0.2	0.00926	12c

Table S4. Refined structure information of the HE-NASICON electrode at 4.5 V.

Crystal phase: trigonal, R-3c (S.G.); a=8.52529 Å, c=21.8675 Å, V=1379.38 Å^3						
Atom	x	y	z	Occ.	Uiso.	Wyckoff. Position
O1	0.0168	0.1775	0.188636	1	0.00100	36f
O2	0.2225	0.1782	0.07445	1	0.20597	36f
P	0.2944	0	0.2500	1	0.00100	18e
Na1	0	0	0	0.705	0.07202	6b
Na2	0.6147	0	0.25	0.182	0.05585	18e
Mn	0	0	0.1325	0.2	0.00156	12c
Fe	0	0	0.1325	0.2	0.00156	12c
V	0	0	0.1325	0.2	0.00156	12c
Ti	0	0	0.1325	0.2	0.00156	12c
Cr	0	0	0.1325	0.2	0.00156	12c

Reference

[1] Y. J. Guo, P. F. Wang, Y. B. Niu, X. D. Zhang, Q. Li, X. Yu, M. Fan, W. P. Chen, Y. Yu, X. Liu, Q. Meng, S. Xin, Y. X. Yin, Y. G. Guo, *Nat. Commun.* **2021**, *12*, 5267.

[2] Q. Zheng, H. Yi, W. Liu, X. Li, H. Zhang, *Electrochim. Acta* **2017**, *238*, 288.

[3] Y. Liu, Y. Zhou, J. Zhang, Y. Xia, T. Chen, S. Zhang, *ACS Sus. Chem. Eng.* **2016**, *5*, 1306.

[4] Y. Zhao, X. Gao, H. Gao, H. Jin, J. B. Goodenough, *Adv. Funct. Mater.* **2020**, *30*, 1908680.

[5] H. Li, T. Jin, X. Chen, Y. Lai, Z. Zhang, W. Bao, L. Jiao, *Adv. Energy Mater.***2018**, *8*, 1801418.

[6] H. Gao, I. D. Seymour, S. Xin, L. Xue, G. Henkelman, J. B. Goodenough, *J. Am. Chem.l Soc.* **2018**, *140*, 18192.

[7] H. Li, M. Xu, C. Gao, W. Zhang, Z. Zhang, Y. Lai, L. Jiao, *Energy Storage Mater.* **2020**, *26*, 325.

[8] J. Zhang, Y. Liu, X. Zhao, L. He, H. Liu, Y. Song, S. Sun, Q. Li, X. Xing, J. Chen, *Adv. Mater.* **2020**, *32*, e1906348.

[9] D. Wang, X. Bie, Q. Fu, D. Dixon, N. Bramnik, Y. S. Hu, F. Fauth, Y. Wei, H. Ehrenberg, G. Chen, F. Du, *Nat. Commun.* **2017**, *8*, 15888.

[10] Q. Zhu, X. Chang, N. Sun, H. Liu, R. Chen, F. Wu, B. Xu, *J. Mater. Chem. A* **2017**, *5*, 9982.
